# *Arabidopsis* Calmodulin-Like Proteins, CML15 and CML16 Possess Biochemical Properties Distinct from Calmodulin and Show Non-overlapping Tissue Expression Patterns

**DOI:** 10.3389/fpls.2017.02175

**Published:** 2017-12-22

**Authors:** Adenike Ogunrinde, Kim Munro, Alexandra Davidson, Midhat Ubaid, Wayne A. Snedden

**Affiliations:** ^1^Department of Biology, Queen's University, Kingston, ON, Canada; ^2^Protein Function Discovery Laboratory, Department of Biomedical and Medical Sciences, Queen's University, Kingston, ON, Canada

**Keywords:** plant, *Arabidopsis*, calcium signaling, calmodulin, CML, gene expression, protein biochemistry

## Abstract

Calcium ions are used as ubiquitous, key second messengers in cells across eukaryotic taxa. In plants, calcium signal transduction is involved in a wide range of cellular processes from abiotic and biotic stress responses to development and growth. Calcium signals are detected by calcium sensor proteins, of which calmodulin (CaM), is the most evolutionarily conserved and well-studied. These sensors regulate downstream targets to propagate the information in signaling pathways. Plants possess a large family of calcium sensors related to CaM, termed CaM-like (CMLs), that are not found in animals and remain largely unstudied at the structural and functional level. Here, we investigated the biochemical properties and gene promoter activity of two closely related members of the *Arabidopsis* CML family, CML15 and CML16. Biochemical characterization of recombinant CML15 and CML16 indicated that they possess properties consistent with their predicted roles as calcium sensors. In the absence of calcium, CML15 and CML16 display greater intrinsic hydrophobicity than CaM. Both CMLs displayed calcium-dependent and magnesium-independent conformational changes that expose hydrophobic residues, but the degree of hydrophobic exposure was markedly less than that observed for CaM. Isothermal titration calorimetry indicated two and three calcium-binding sites for CML15 and CML16, respectively, with affinities expected to be within a physiological range. Both CML15 and CML16 bound calcium with high affinity in the presence of excess magnesium. Promoter-reporter analysis demonstrated that the *CML16* promoter is active across a range of *Arabidopsis* tissues and developmental stages, whereas the *CML15* promoter activity is very restricted and was observed only in floral tissues, specifically anthers and pollen. Collectively, our data indicate that these CMLs behave biochemically like calcium sensors but with properties distinct from CaM and likely have non-overlapping roles in floral development. We discuss our findings in the broader context of calcium sensors and signaling in *Arabidopsis*.

## Introduction

Cellular signaling has evolved in eukaryotes as a means of detecting and deciphering information and organizing appropriate and timely responses to internal and environmental stimuli. The use of small molecules and ions as second messengers is a highly conserved aspect of signal transduction and information processing in eukaryotes. Although most cells employ a range of second messengers, the divalent metal Ca^2+^ is considered the most universal and ubiquitous as it functions across a diverse range of cellular signaling events (Edel and Kudla, 2015; Marchadier et al., 2016; Edel et al., 2017).

In plants, Ca^2+^ signaling plays important roles in responses to stimuli such as abiotic stress (e.g., drought, salinity, temperature stress), biotic stress (e.g., microbial attack, viral attack), as well as many developmental cues (Ranty et al., 2006; DeFalco et al., 2010). In the standard Ca^2+^ signal transduction model, resting cytosolic Ca^2+^ [Ca^2+^]_cyt_ is maintained at nM (~100 nM) levels through active transport into Ca^2+^ stores (e.g., central vacuole) or out of the cell into the apoplast, thereby creating Ca^2+^ gradients several orders of magnitude (Steinhorst and Kudla, 2014). Complex patterns of cytosolic Ca^2+^ influx and efflux, controlled via channel and pump activities, are evoked in response to various stimuli. The Ca^2+^-signature hypothesis posits that these spatial-temporal patterns of Ca^2+^ signals encode information about the nature of the evoking stimulus (Berridge et al., 2003; Bender and Snedden, 2013). A corollary of this model is that different stimuli lead to distinct Ca^2+^ signatures that can be decoded by mechanisms within the cell. Others have suggested that Ca^2+^ signals act less like coded signatures and more like a switch or lock-and-key in combination with additional messengers (Scrase-Field and Knight, 2003; Plieth, 2016). These models are not mutually exclusive and recognize that, regardless of the nature of the signal, specific Ca^2+^-binding proteins must serve as sensors to detect changes in Ca^2+^ levels and function to activate appropriate signaling cascades. Calmodulin (CaM) is the canonical Ca^2+^ sensor and represents one of the most evolutionarily-conserved eukaryotic proteins. CaM is a sensor relay protein in that it lacks inherent catalytic activity and functions instead through the direct binding and regulation of downstream targets including ion-pumps and channels, transcription factors, metabolic enzymes, cytoskeletal proteins, and a wide range of other proteins (Ikura and Ames, 2006; Bender and Snedden, 2013). CaM is a small (~17 kDa) protein that has been well-characterized at the structural level, consisting of N- and C-terminal globular regions separated by a flexible central helix, giving rise to what has been described as a dumbbell-shaped molecule. Each globular region of CaM contains a pair of Ca^2+^-binding EF-hand motifs. A classic EF-hand consists of a 29-residue helix-loop-helix structure (Strynadka and James, 1989). Seven oxygen-containing groups, five in the EF loop (at positions 1, 3, 5, 7, and 9) and two donated by the glutamate outside of the loop (at position 12), are responsible for coordinating a Ca^2+^ ion in a pentagonal bipyramidal arrangement (Strynadka and James, 1989; Falke et al., 1994; Gifford et al., 2007; Grabarek, 2011). Residues 1, 3, 5, 7, 9, and 12 are referred to as X, Y, Z, –*Y*, –*X*, and –*Z*, respectively. Ca^2+^-binding to the EF-hands of sensors triggers reversible conformational changes in tertiary structure that lead to the exposure of hydrophobic residues. These hydrophobic regions facilitate interactions with downstream effector proteins, which in turn elicit a physiological response to the initial Ca^2+^ signal (Yamniuk et al., 2004). In CaM, as in most EF-hand Ca^2+^ sensors, EF-hands are paired and exhibit positive cooperativity with each hand displaying an increased affinity for Ca^2+^ after the partner has bound Ca^2+^ (Akke et al., 1991; Gifford et al., 2007).

In the genetic model plant, *Arabidopsis*, there are about 250 EF-hand proteins encoded in the genome (Day et al., 2002). Similarly, 262 EF-hand proteins are predicted in soybean, suggesting such complexity is found throughout plant taxa (Zeng et al., 2017). In addition to CaMs, plants possess three additional large families of Ca^2+^ sensors: Ca^2+^-dependent protein kinases (CPKs), calcineurin B-like proteins (CBLs), and CMLs (DeFalco et al., 2010; Ranty et al., 2016). CaMs, CBLs, and CMLs fall into the class of sensor-relay proteins, whereas CPKs are considered sensor responders as they possess both Ca^2+^ binding and kinase activities. Remarkably, although CaM is highly conserved throughout all eukaryotes, CBLs, CPKs, and CMLs are found only in plants and some protists (DeFalco et al., 2010; Beckmann et al., 2016). The *Arabidopsis* genome encodes 7 *CaM* genes (*AtCaM1-AtCaM7*), with identical isoforms and 4 distinct but highly conserved isoforms that differ by up to 4 amino acids (McCormack and Braam, 2003). In addition, *Arabidopsis* possesses 50 CML proteins (AtCMLs) that range from 16 to 75% sequence identity with conserved CaM (e.g., AtCaM2) and are arranged into 9 phylogenetic subgroups (McCormack and Braam, 2003). This large diversity of CMLs is seen across plant taxa (Boonburapong and Buaboocha, 2007; Zhu et al., 2015). Like CaM, these CMLs are thought to localize predominantly to cytosolic and nuclear compartments where Ca^2+^ signals are important (McCormack and Braam, 2003; DeFalco et al., 2010), although CML30 and CML3 have been observed in mitochondria and peroxisomes, respectively (Chigri et al., 2012).

Structurally, plant CMLs resemble CaM and are predicted to possess EF-hands, and no other known functional domains. *Arabidopsis* CaMs have 149 amino acids and possess 4 EF-hands, whereas AtCMLs range from 80 to 330 amino acids and possess from 2 to 4 predicted EF-hands with CML12 (6 EF-hands) deviating from this paradigm (McCormack and Braam, 2003). The evolutionary divergence of CMLs from CaM, their conservation across plant taxa, and the fact that CMLs are restricted to plants and some protists, suggests that CMLs play unique roles in plant Ca^2+^ signaling pathways. Although a number of recent reports have implicated plant CMLs in processes ranging from biotic and abiotic stress response to development, the vast majority of CMLs in *Arabidopsis*, and in plants in general, remain unstudied.

In order to understand how plants coordinate physiological responses from such a common second messenger, a comprehensive understanding of the Ca^2+^ sensors unique to plants is required. To this end, we have been systematically studying the biochemical and physiological properties of *Arabidopsis* CMLs including CML42, CML43, CML37, CML38, CML39 (Chiasson et al., 2005; Vanderbeld and Snedden, 2007; Dobney et al., 2009; Bender et al., 2013, 2014; Scholz et al., 2014). Here, were describe an investigation into two putative Ca^2+^ sensors of unknown function, CML15 and CML16, which are two closely-related paralogs (73.9% identity) from *Arabidopsis* CML subfamily four. We discuss their distinct biochemical properties and gene promoter activity patterns in light of Ca^2+^ signaling in different tissues and developmental contexts.

## Materials and methods

### Plant material and growth conditions

All experiments were performed with Columbia (Col-0) accession *Arabidopsis thaliana*, referred to as *Arabidopsis*. All *Arabidopsis* seeds sown to soil were stratified at 4°C for 3 days in the dark, before transfer to a growth incubator set at a 16-h light (50 μmol/m^2^s)/8-h dark photoperiod at 22°C and at 70% relative humidity. Plants were supplemented with 1 g/L 20-20-20 (N-P-K) fertilizer every 3 weeks until senescence. In some experiments, seeds were surface sterilized as described (Bender et al., 2013) prior to plating and stratification on sterile petri dishes with 0.5X Murashige and Skoog (MS) medium (Murashige and Skoog, 1962; Caisson Laboratories, Inc.) supplemented with 0.8% (w/v) agar followed by cold-stratification as described above. Transgenic lines for *CML* promoter-β-glucuronidase (GUS) reporter activity analysis (*CMLpro::GUS* plants) were selected on media containing kanamycin as described (Bender et al., 2013) supplemented with 1% sucrose. Transgenic *Arabidopsis CML15pro::GUS* and *CML16pro::GUS* transgenic lines were grown for two subsequent generations to obtain homozygous lines for analyses of *CMLpro::GUS* reporter activity. Preliminary analysis showed comparable and reliable qualitative patterns of expression among several lines tested and *CML15pro::GUS* lines 1A and 3C were used to assay *CML15pro::GUS* reporter activity, and lines 12A and 14B were used to assay *CML16pro::GUS* reporter activity.

### Plasmid construct design and cDNA cloning

The protein-coding regions of *CML15* and *CML16*, both intronless genes, were cloned into the pET21b vector (Novagen) for recombinant expression in *Escherichia coli* (*E. coli*). The genomic region from the ATG translation start site to the TGA translation stop site of *CML15* (AT1G18530), and *CML16* (ATG3G25600), were amplified by PCR and independently subcloned into the pET21b expression vector (Novagen) using the CML-F and CML-R primers described in Supplementary Table 1. The evolutionarily-conserved CaM, CaM81 from petunia, which is 100% identical to *Arabidopsis* CaM2, and cloned into a pET5a vector (Novagen), was a gift from Hillel Fromm (Tel Aviv University). All constructs were transformed into *E. coli* strain BL21 (DE3) RPRIL or PLysS (Novagen), for the expression of recombinant CML15 and CML16, or CaM81, respectively.

### Cloning the CML15 promoter and CML16 promoter into pBI101

The putative promoter regions of *CML15* and *CML16* were amplified from genomic DNA using CMLPRO-F and CMLPR-R primers (Supplementary Table 1) and cloned into the binary vector pBI101 to control the expression of the gene-reporter, β-glucuronidase (GUS) (Jefferson et al., 1987). The regions of genomic DNA upstream of *CML15* (*CML15pro*) and *CML16* (*CML16pro*) from the stop site of the nearest upstream gene to the ATG start translation site of each respective *CML* were subcloned into the binary vector pBI101 to yield *CML15pro::GUS* and *CML16pro::GUS* constructs. *CML15pro* (591 bp) and *CML16pro* (700 bp) are referred to as the putative promoter regions of *CML15* and *CML16*, respectively. Each construct was confirmed by sequencing (SickKids, Toronto) and chemically transformed into *Agrobacterium tumefaciens* strain GV3101 for stable transformation into *Arabidopsis* via the floral dip method (Clough and Bent, 1998).

### Histochemical GUS assays

Transgenic *Arabidopsis* for *CMLpro::GUS* were stained for GUS activity, as a proxy for *CML*-promoter activity, using the protocol essentially described (Jefferson et al., 1987; Vanderbeld and Snedden, 2007) with the following exceptions. Plant tissue was fixed in 90% acetone at 4°C for 1 h, then rinsed in double-deionized water 3 times before incubation in the GUS-staining solution (100 mM NaH_2_PO_4_, 100 mM Na_2_HPO_4_, pH 7.0, containing 10 mM EGTA and 0.1% (v/v) TritonX-100) with the substrate 5-bromo-4-chloro-3-indolyl-β-D-glucuronide (X-gluc) added to 0.1 mg/ml. Tissues were incubated for ~18 h at 37°C with shaking, and were subsequently rinsed several times with 70% ethanol. Stained tissue was then stored in 70% ethanol. Developmental histochemical GUS assays were performed on 1-, 7-, 10-, 22-, 25-day old, and fully mature *Arabidopsis* tissues. The representative developmental stages were selected based on Boyes et al. (2001).

### Fluorometric GUS assays

Transgenic *Arabidopsis* for *CMLpro::GUS* analyses were quantitatively assayed for GUS activity as described (Vanderbeld and Snedden, 2007). Seedling, mature leaf, and mature floral tissues were harvested in 1.5-mL microfuge tubes and snap frozen with liquid nitrogen. Frozen tissue was ground with a micropestle in 500 μL of QB buffer (0.1 M KPO_4_, 1 mM EDTA, 0.1% (v/v) Triton X-100, 10% (v/v) glycerol, and 0.1% (w/v) N-lauryl sarcosine sodium salt) in the presence of about 100 mL sterile sand and 100 mL 1.5 mm metal beads to facilitate grinding. The ground tissue was further homogenized using the Next Advanced Bullet Blender® at high speed for 5 min, centrifuged at 10,000 × g for 10 min at 4°C, and the resulting supernatant was assayed for protein concentration using the Bradford reagent (BioShop) as described (Bradford, 1976). Protein extract (50 μL) was added to 450 μL QB buffer supplemented with 1 mM DTT and 0.5 mg/mL 4-Methylumbelliferyl-β-D-glucuronide (MUG; BioShop) and samples were incubated at 37°C for 2 h after which time reactions were quenched with 400 μL of 0.2 M Na_2_CO_3_. For quantitative analysis of GUS activity, triplicate samples were assayed in a black Fluotrac-200 96-well microplate and the emission fluorescence of 4-MU (4-methylumbelliferone) at 455 nm was measured after excitation with wavelength of 365 nm using a Spectramax Gemini XS spectrophotometer. Average fluorescence values were subsequently converted to units of specific activity (pmoles 4-MU/μg protein/min).

### Recombinant protein expression and purification

Preparation of recombinant CaM and CMLs was essentially as described (Zielinski, 2002). *E. coli* cultures (10 mL) expressing CaM81, CML15, or CML16, respectively, were grown overnight (~10 h) at 37°C. The 10-mL cultures were used to inoculate 1–1.25 L of LB media, until an OD_600_ = 0.6–0.8 was reached, at which point isopropyl-β-D-thiogalactopyranoside (IPTG) was added (0.5 mM) to induce recombinant protein expression. After 4–6 h *E. coli* cells were isolated by centrifugation and suspended in a lysis buffer (50 mM Tris-Cl, pH 7.5, 1.5 mM EDTA, 1 mM DTT, 1 mM benzamidine, 1 mM PMSF) at 1/20 culture volume and were lysed using a French® Pressure Cell Press (Thermo Scientific). Recombinant proteins were purified by Ca^2+^-dependent phenyl-sepharose column chromatography, essentially as described (Zielinski, 2002) with the following exceptions. The binding of CaM81, CML15, and CML16 (in CaM-binding buffer: 50 mM Tris-Cl, pH7.5) to phenyl-sepharose was performed in the presence of CaM-binding buffer containing 2 mM CaCl_2_, followed by 2–5 column-volume washes of CaM-binding buffer with 200 mM NaCl, 2–5 column-volume washes of CaM-binding buffer with 400 mM NaCl, prior to elution of adsorbed protein with CaM-binding buffer containing 2 mM EGTA. Protein was eluted over 8 fractions in volumes of elution buffer equivalent to the bed volume of the resin (~1–2 mL). The eluted fractions were resolved on 17% SDS-PAGE gels which were then stained with Coomassie Brilliant Blue (R250) and subsequently destained [50% ddH_2_0 (v/v), 40% MeOH (v/v), and 10% glacial acetic acid (v/v)] to evaluate purity. Insufficiently pure fractions were pooled, and re-purified over phenyl-sepharose 1–2 more times using the method described above. In order to achieve near-homogeneity of recombinant CML16, following phenyl-sepharose chromatography, pooled CML16 samples were resolved by fast protein liquid chromatography (FPLC) using a Superdex-75 gel-filtration column to eliminate contaminating *E. coli* proteins of ~37 and ~65 kD. Prior to FPLC, the gel filtration column was pre-equilibrated with 2 mM EGTA (in 50 mM Tris-Cl, pH 7.5). Fractions (2 mL) were collected and analyzed for purity by SDS-PAGE and Commassie staining. Pure recombinant CMLs were concentrated using 4,000- or 10,000-kD cutoff Amicon® Ultra-4 spin-column centrifugal filters (Millipore). Protein samples were then dialyzed overnight into various buffers suited for specific biochemical analyses as described below. In an effort to minimize Ca^2+^ contamination of samples, for all biochemical analyses, milli-Q (Millipore Sigma) purified water was used for all solutions and for rinsing of glassware. Dialysis tubing was treated with 1 mM EDTA and 1 mM EGTA, followed by extensive rinsing with milli-Q purified water.

### ANS fluorescence spectroscopy

The hydrophobic properties of recombinant CaM, CML15 and CML16 were monitored using ANS (8-anilinonapthalene-1-sulfonic acid) fluorescence emission spectroscopy using an excitation wavelength of 380 nm, and scanning emission spectra 430–600 nm, performed essentially as described (Dobney et al., 2009). CaM and CML samples were dialyzed overnight against 1 L of ANS buffer (10 mM Tris-CL, pH 7.5, containing 1 mM DTT, and 100 mM KCl). The fluorescence emission of 15 μM CML15 and 15 μM CML16 was monitored using 250 μM ANS in ANS buffer. Spectra were recorded at room temperature (22°C) under various conditions of CaCl_2_, MgCl_2_, EGTA, or Na_3_C_6_H_6_O_7_ in ANS buffer, as described in Figure Legends. Ca^2+^-CaM81 (15 μM) was monitored as a positive control for Ca^2+^-induced hydrophobicity. ANS background fluorescence was monitored and subtracted from the fluorescence readings of the protein-ANS buffer conditions. Average fold-increases in peak florescence emission of ANS in the presence of 15 μM CML15, 15 μM CML16 or 15 μM CaM81, in the presence of 1 mM CaCl_2_ + 5 mM MgCl_2_ vs. 1 mM MgCl_2_, were calculated as the fold-increase in fluorescence due to Ca^2+^-binding. Fold-induction was averaged over four independent biological replicates with three technical replicates per condition.

### Phenyl-sepharose chromatography of recombinant CMLs under various conditions

In order to assess the dependence of hydrophobic exposure of CMLs on Ca^2+^ binding, the ability of CML15 and CML16 to bind to, and elute from, phenyl-sepharose under various ionic conditions was tested. Protein samples and the associated wash buffers were adjusted to final concentrations of either 1 mM CaCl_2_, or 1 mM MgCl_2_, or 1 mM CaCl_2_ + 1 mM MgCl_2_, or 1 mM EGTA, in 50 mM Tris-Cl, pH7.5, respectively, prior to phenyl-sepharose binding. Proteins were subjected to hydrophobic interaction column chromatography, essentially as described above. Post phenyl-sepharose binding, columns were washed (10 mL) using the same buffer and conditions as noted above but with the addition 200 mM KCl, followed a second wash (10 mL) which included 400 mM KCl. All column elutions were performed using 50 mM Tris-Cl, pH7.5 containing either 2 mM EGTA (for samples loaded with 1 mM CaCl_2_), or 3 mM EDTA (for samples loaded with 1 mM MgCl_2_), or 2 mM EGTA + 3 mM EDTA (for samples loaded with 1 mM CaCl_2_ and 1 mM MgCl_2_), or 1 mM EGTA (for samples loaded with 1 mM EGTA). Samples of the unbound protein (flow-through), washes, elutions, and the pre-column protein samples were resolved by SDS-PAGE and analyzed by staining with Coomassie Brilliant blue (R250).

### Circular dichroism (CD) spectroscopy

Far-UV CD spectroscopy was performed to evaluate the secondary structure of recombinant CML15 and CML16 as described (Bender et al., 2013). Spectra were collected and analyzed at the Queen's Protein Function Discovery facility using a Chirascan CD spectrophotometer. Samples of CML15 and CML16 were independently dialysed into 2 mM Tris-Cl (pH 7.5), quantitated (45 μM and 30 μM, respectively), and analyzed by CD spectroscopy, using a cylindrical quartz cuvette with a pathlength of 0.1 mm. Spectra for both CML15 and CML16 were initially obtained in the presence of 1 mM EGTA, 1 mM CaCl_2_, or 1 mM MgCl_2_. To confirm that the spectral changes under these conditions represented either complete Ca^2+^ chelation or Ca^2+^ saturation, samples were also analyzed in the presence of 2 mM EGTA or 2 mM CaCl_2_. For each experimental condition, spectra were obtained as an average of 6 raw scans which were subsequently reference-corrected vs. buffer-only scans and adjusted for molar concentration. Average, corrected CD spectra obtained for each experimental condition were deconvoluted using both the OLIS GlobalWorks and CDNN deconvolution software to estimate protein secondary structural composition.

### Isothermal titration calorimetry (ITC)

ITC experiments were performed using a MicroCal VP-ITC microcalorimeter to determine the affinities of Ca^2+^ for the EF-hand domains of CML15 and CML16, and to examine the effect of Mg^2+^ on these Ca^2+^-binding events. Prior to ITC titrations, CML15 and CML16 samples were dialysed into 20 mM HEPES (pH 7.5) containing 100 mM KCl, either with or without 5 mM MgCl_2_. Samples of CML15 or CML16 were independently titrated at 30°C with twenty-nine 10 μL injections of either 600 μM CaCl_2_ or 600 μM MgCl_2_ at 6-min intervals. For each experimental condition, titrations were performed in triplicate. Concentrations of CML15 and CML16 used for data analysis of isotherms (see Figure Legends) were determined by amino-acid analysis (SickKids Hospital, Toronto). Origin 7.0 software (MicroCal) was used to determine the enthalpies (ΔH), association constants (K_*a*_), binding entropies (ΔS), and stoichiometries of CML15 and CML16 with respect to Ca^2+^ and Mg^2+^ binding. K_*d*_ values were determined using 1/K_*a*._

### Molecular modeling of CMLs

The protein homology/analogy recognition engine (Phyre2.0) website (http://www.sbg.bio.ic.ac.uk/phyre2) was used to predict structural models CML15 and CML16 based on the highest-ranked homologous model in the database using default parameters (Kelley et al., 2015). Model images were generated using the PyMOL molecular graphics system, version 1.9. Schrodinger, LLC (http://www.pymol.org). Grand average of hydropathy (GRAVY) scores were determined using the ProtParam tool from ExPasy online bioinformatics portal (www.expasy.org, Artimo et al., 2012).

## Results

### Sequence comparison of CML15, CML16, and conserved CaM

**Figure 1:**
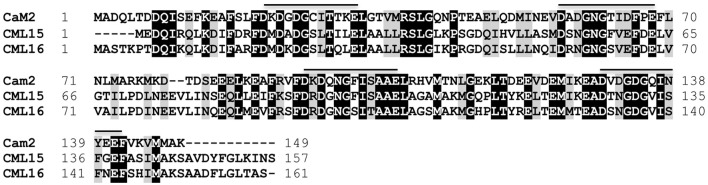
Amino-acid sequence comparison of *Arabidopsis* CML15 (accession Q9FZ75) and CML16 (accession Q9LI84) to the evolutionarily-conserved CaM, CaM2 (accession NP_850344). Identical and conserved residues are shaded in black and gray, respectively, and alignment gaps are indicated by dashes. Positions of Ca^2+^-binding EF-hands for CaM2, and the corresponding regions of the CMLs, are indicated with a line. Residue positions are presented to the left and right of each sequence. Sequences were aligned using Clustal Omega (Sievers et al., 2011).

CML15 and CML16 are closely related paralogs showing 74% sequence identity with each other and 39.5% identity with the evolutionarily-conserved CaM, AtCaM2 (Figure 1). CML15 and CML16, along with CML17 and CML18 comprise the four members of subgroup IV in the Arabidopsis CaM/CML phylogeny (McCormack et al., 2005). A recent reorganization of the Arabidopsis phylogeny places CML15 and CML16 in subgroup II (Zhu et al., 2015). Orthologs of both these CMLs are found across plant taxa (Zhu et al., 2015; Zeng et al., 2017). Although CML15 and CML16 are predicted to possess 4 EF-hand motifs, variations within the Ca^2+^-binding loops and adjacent regions are present when compared to CaM2. The backbone glycine in position 6 (Gly_6_), and Glu_12_, are conserved across the four EF-loops of CML15 and CML16. Conversely, across all four EF-loops of the two CMLs and CaM, the residues found in positions 7 and 9 are variable. This holds true for the residue at position 8 as well, which though variable, is consistently hydrophobic. EF-loop I of CML15 and CML16 do not differ substantially from EF-loop I of AtCaM2 with respect to the residues typically involved in forming the Ca^2+^-coordination sphere. Residues 1, 3, 5, 9, and 12 of EF-loop I are identical to those of AtCaM2. The third residue of EF-hand II and IV, however, for both CML15 and CML16, has an asparagine (Asn) substitution rather than an aspartate typically found in this position (Gifford et al., 2007). Finally, position 9 of EF-loop III of CML15, CML16, and GmCaM4, and position 9 of EF-loop IV of CML15 and CML16, is occupied by a serine (Ser_9_). This is unlike AtCaM2, which has a threonine (Thr_9_) in EF-loop III and an Asp_9_ in EF-loop IV. This range of substitutions in the EF hands of these CMLs suggests their Ca^2+^-binding properties are likely distinct from those of CaM.

### Calcium-binding properties of CML15 and CML16

**Figure 2:**
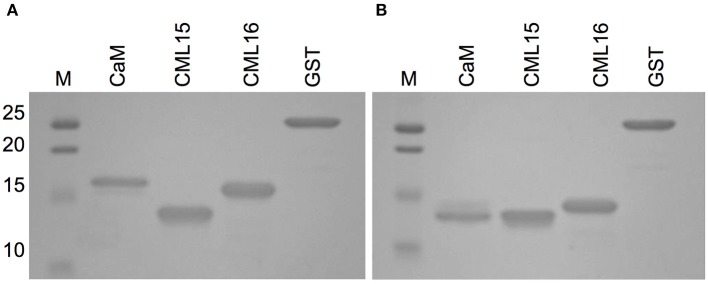
Ca^2+^-dependent electrophoretic mobility analysis of recombinant CML15 and CML16 using SDS-PAGE in the presence of **(A)** 5 mM EGTA or **(B)** 5 mM CaCl_2_. Recombinant CaM served as a positive control for electrophoretic mobility shifts in the presence of Ca^2+^ and purified recombinant glutathione S-transferase (GST) served as the negative control. SDS-PAGE gels were stained with Coomassie Brilliant Blue R250. Molecular weight markers (MW, kDa) are indicated on the left. Representative images from a minimum of three experimental replicates are presented.

The increased electrophoretic mobility of CaM in the presence of Ca^2+^ is a hallmark property that has been used as a comparison when evaluating various putative Ca^2+^ sensors for their ability to bind Ca^2+^ (Garrigos et al., 1991; Dobney et al., 2009). Although both CML15 and CML16 displayed shifts in electrophoretic mobility in the presence of Ca^2+^, the relative mobility shift of CML16 was more pronounced than that observed for CML15 which was barely perceptible (Figure 2).

**Figure 3:**
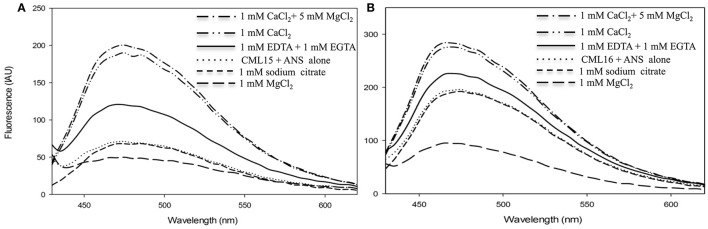
Ca^2+^- and Mg^2+^-induced changes in exposed hydrophobicity of recombinant CML15 and CML16 as demonstrated by ANS fluorescence. Fluorometric scans (430-600 nm) were recorded following the addition of 15 μM **(A)** CML15 or **(B)** CML16 to 250 μM ANS where all sample conditions used ANS buffer (10 mM Tris-Cl pH 7.5, 100 mM KCl, and 1 mM DTT). The *y*-axis depicts fluorescence in international arbitrary units (IAU). Fluorescence values were analyzed relative to a protein-free ANS control sample that was subtracted from the data as background. Apo-CML (CML + ANS alone) scans represent baseline CML hydrophobicity. Scans were recorded under the conditions noted in the figure panels by adding various compounds to apo-CML. Each data set is representative of five experimental replicates.

An additional property of CaM and related Ca^2+^ sensors are reversible conformation changes that expose hydrophobic regions upon Ca^2+^ binding (Yamniuk et al., 2004; Ikura and Ames, 2006). We investigated CML15 and CML16 for Ca^2+^-mediated increases in hydrophobic exposure using ANS-based fluorescence and phenyl-sepharose chromatography. ANS-fluorescence spectroscopy was conducted to assess the nature of exposed hydrophobicity for CML15 and CML16 under various *in vitro* conditions. A blue shift and associated increase in the intensity of the fluorescence emission of ANS was observed in the presence of Ca^2+^-CML15 and Ca^2+^-CML16 (Figure 3) compared to protein in ANS buffer alone. The greatest relative blue-shifts in emission spectra were observed for CML15 and CML16 in the presence of 1 mM CaCl_2_ or in the presence of both 1 mM CaCl_2_ and 5 mM MgCl_2_. Only a very weak increase in ANS fluorescence was observed for CML15 or CML16 in the presence of 1 mM MgCl_2_ alone. There was a 4.05 ± 0.31-fold increase in ANS fluorescence for Ca^2+^-CML15 (in a 5 mM MgCl_2_ background) compared to Mg^2+^-CML15. Likewise, there was a 2.44 ± 0.23-fold increase in ANS fluorescence for Ca^2+^-CML16 (in a 5 mM MgCl_2_ background) compared to Mg^2+^-CML16. Based on their amino acid compositions, we note that both CML15 and CML16 have grand average of hydrophobicity (GRAVY) scores of 0.009 and −0.171, respectively, which are higher than that for CaM at −0.619 (Supplementary Figure 3). However, CaM showed a much greater increase in exposed hydrophobicity upon binding Ca^2+^ than either CML15 or CML16 (Supplementary Figure 1). Interestingly, there were slight increases in ANS fluorescence, suggesting increased hydrophobicity of CML15 and CML16, in the presence of 1 mM EGTA, even in the absence of Ca^2+^ or with 1 mM EDTA (in the absence of Mg^2+^) relative to protein in ANS buffer alone or in the presence of 1 mM MgCl_2_ (Figure 3). However, ANS fluorescence of CML15 and CML16 in the presence of 1 mM Na_3_C_6_H_5_O_7_ (trisodium citrate), another Ca^2+^-chelator, was similar to that of CML15 and CML16 in ANS buffer (Figure 3). Collectively, these data indicate that CML15 and CML16 possess intrinsic hydrophobicity in the absence of Ca^2+^ or Mg^2+^, and that they display a marked increase in hydrophobic exposure in the presence of Ca^2+^. In addition, CML16 displays greater intrinsic hydrophobicity than CML15 at peak fluorescence emission. These data also show that EGTA and/or EDTA have a positive effect on the hydrophobic exposure of CML15 and CML16 that is less than that induced by Ca^2+^, but greater than their intrinsic hydrophobic exposure. For both CML15 and CML16, the increase in exposed hydrophobicity in response to Ca^2+^ binding is reminiscent of CaM but the magnitude of change and the high level of intrinsic hydrophobicity in the absence of Ca^2+^ are quite different than observed for CaM (Supplementary Figure 1), indicating unique structural properties for these CMLs.

For comparison with our ANS-based data, we further explored the Ca^2+^- and Mg^2+^-mediated hydrophobic properties of CML15 and CML16 using phenyl-sepharose chromatography under various *in vitro* conditions (Supplementary Figure 2). Both CMLs bound to phenyl-sepharose in the presence of 1 mM CaCl_2_ and in the presence of 1 mM CaCl_2_ + 1 mM MgCl_2_, however, neither CML bound in the presence of 1 mM MgCl_2_ alone. Interestingly, there was weak binding of CML15 and CML16 to phenyl-sepharose in the presence of 1 mM EGTA (i.e., absence of Ca^2+^), and a slow elution during column washing with 0.2 and 0.4 M KCl prior to elution with EGTA. Collectively, these data suggest that most of the hydrophobic exposure of these CMLs is triggered by Ca^2+^ binding but there is also a notable degree of intrinsic hydrophobicity present in the absence of Ca^2+^, observations consistent with the ANS fluorescent data (Figure 3).

**Figure 4:**
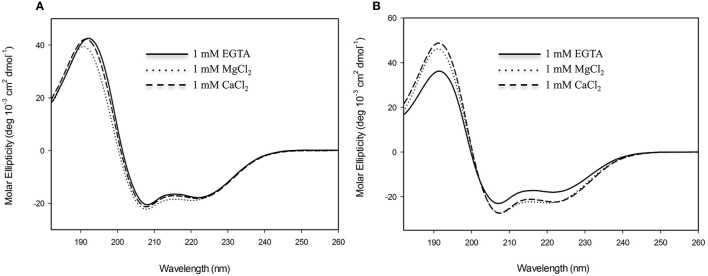
Far-UV circular-dichroism (CD) spectroscopy of recombinant **(A)** CML15 and **(B)** CML16. Proteins were analyzed in 2 mM Tris-Cl (pH 7.5) in the presence of 1 mM EGTA, 1 mM CaCl_2_, or 1 mM MgCl_2_ as noted in figure panels. Far-UV CD spectra (180–260 nm) were collected at room temperature. Each spectrum is representative of 6 scans and is presented in units of molar ellipticity. CML15 and CML16 were analyzed at final concentrations of 45 μM and 30 μM, respectively.

In order to evaluate the secondary structural characteristics of CML15 and CML16 in the apo-form, and in the presence of Mg^2+^ and Ca^2+^, we performed far-UV CD spectroscopy (Figure 4). CD spectra of CML15 and CML16 show that both proteins are structured, each presenting predominantly α-helical content in the absence of any divalent cation, as indicated by the peaks of molar ellipticity at 190 nm and troughs at 208 and 222 nm, respectively. CML15 showed a slight (3–6%) decrease in α-helical content in the presence of saturating Ca^2+^ (1 mM CaCl_2_), whereas CML16 showed ~8–15% increase in α-helical content in the presence of 1 mM CaCl_2_. Interestingly, Mg^2+^-CML16 displayed similar CD spectra to that of Ca^2+^-CML16, suggesting that both Ca^2+^ and Mg^2+^ binding elicits structural increases in the α-helical content of CML16.

**Figure 5:**
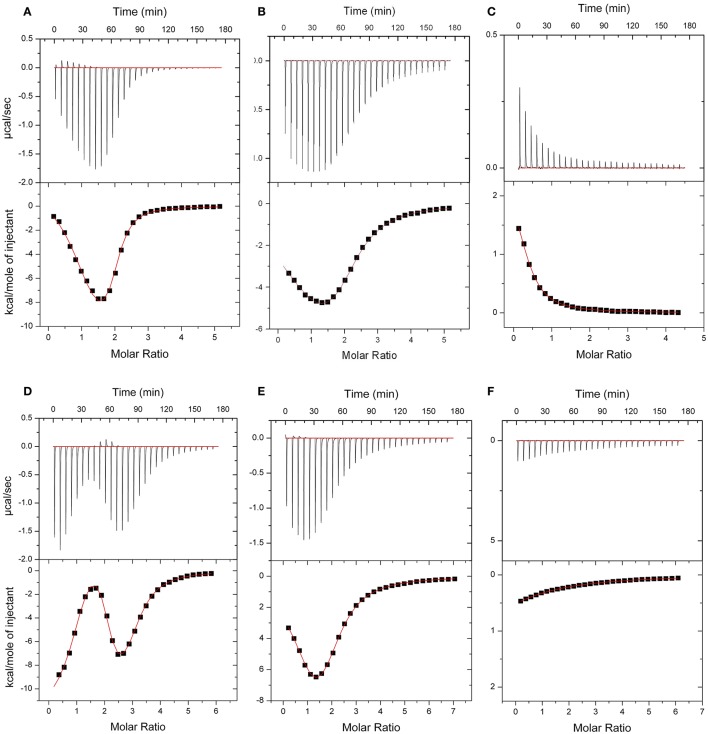
ITC analysis of Ca^2+^ and Mg^2+^ binding to CML15 **(A–C)** and CML16 **(D–F)**. Representative isotherms are presented for Ca^2+^ titration into CML samples in either the absence **(A,D)** or presence **(B,E)** of 5 mM Mg^2+^. Also presented are Mg^2+^ titrations in a Ca^2+^-free background **(C,F)** for CML15 and CML16, respectively. Binding was monitored at 30°C in 25 mM HEPES, 100 mM KCl, pH 7.5. The upper panels show raw data from calorimetric titrations, using 5 μL injections, of recombinant CMLs with 600 μM CaCl_2_ or 600 μM MgCl_2_ as noted above. The lower panels present the corresponding integrated binding isotherm modeled for two Ca^2+^-binding events for CML15 and three Ca^2+^-binding events for CML16. Each isotherm is representative of a minimum of three technical replicates. Protein was used at the following concentrations (**A** = 26 μM, **B** = 26 μM, **C** = 31 μM, **D** = 23 μM, **E** = 19 μM, **F** = 23 μM).

The energetics of Ca^2+^ (and Mg^2+^) binding to recombinant CML15 and CML16 were studied by ITC (Figure 5). Raw ITC data representing the endothermic and/or exothermic nature of divalent cation binding events were used to generate Wiseman ITC plots which in turn allowed for estimation of the stochiometries and affinities for Ca^2+^ or Mg^2+^ binding, as well as the relative entropic and enthalpic contributions of each binding event (Table 1). Amino-acid sequence data indicates that both CMLs are predicted to have four EF-hands (Figure 1; McCormack and Braam, 2003), however, ITC analysis suggests both CMLs may only have a subset of sites that are active. Data were fitted using the Origin 7.0 software to give stochiometries of two Ca^2+^-binding sites for CML15 and three Ca^2+^-binding sites for CML16. Analysis of ITC data predicted Ca^2+^-binding affinities for CML15 of 0.22 and 1.23 μM for site 1 and site 2, respectively, in the absence of Mg^2+^. These affinities are reduced to 1.56 and 7.25 μM when excess Mg^2+^ is present (Table 1). In either the presence or absence of Mg^2+^, thermodynamic data (Table 1) suggests that favorable entropy drives site-1 Ca^2+^ binding whereas site-2 binding is driven predominantly by favorable enthalpy (Figures 5A–C). Although it is not possible to determine stochiometries from the Mg^2+^ titration, a macroscopic binding constant of 6.95 μM for Mg^2+^ was estimated (Table 1). Unlike the Ca^2+^-binding event, which is exothermic, Mg^2+^ binding to CML15 (Figure 5C) is endothermic; consequently, entropy is the predominant driving force for Mg^2+^ binding.

**Table 1:** Thermodynamic parameters and dissociation values for Ca^2+^ binding to CML15 and CML16.

	**Mg**^**2+**^**-free background, Ca**^**2+**^ **titration**			**5 mM Mg**^**2+**^ **background, Ca**^**2+**^ **titration**		
	**K*_*d*_* (μM)^a^**	**ΔH (kJ mol^−1^)**	**ΔS (J K^−1^ mol^−1^)**	**K*_*d*_* (μM)**	**ΔH (kJ mol^−1^)**	**ΔS (J K^−1^ mol^−1^)**
**CML15**						
Site 1	0.22 ± 0.06	7.30 ± 3.84	151.46	1.56 ± 0.62	−9.12 ± 3.18	−102.38
Site 2	1.23 ± 0.16	−60.17 ± 1.04	−85.35	7.25 ± 0.51	48.99 ± 11.34	147.19
**CML16**						
Site 1	0.03 ± 0.01	−42.38 ± 0.83	5.77	0.91 ± 0.28	−7.49 ± 1.98	90.79
Site 2	0.21 ± 0.02	11.93 ± 1.05	167.07	7.09 ± 0.59	−69.12 ± 13.13	−129.29
Site 3	3.76 ± 0.23	−55.06 ± 0.82	−77.82	ND		

a *Mean ± SE values*.

For CML16, in a Mg^2+^-free background, we observed three Ca^2+^-binding sites ranging in affinity from 0.03 μM up to 3.76 μM. For site-1, both entropic and enthalpic values were favorable, whereas the remaining sites were driven either by favorable entropy (site-2) or enthalpy (site-3). In contrast, in the presence of Mg^2+^, only two Ca^2+^-binding sites were observed with markedly reduced affinities of 0.91 and 7.09 μM, respectively. When Mg^2+^ is present, favorable entropy and enthalpy drive Ca^2+^ binding to the higher-affinity site, whereas the weaker site is enthalpically driven. In the absence of Ca^2+^, CML16 displays a macroscopic binding constant for Mg^2+^ of 54 μM (Figure 5F). In contrast to CML15, Mg^2+^ binding appears to be both entropically and enthapically driven.

### *CML15*- and *CML16*-promoter activity in *Arabidopsis* tissues

**Figure 6:**
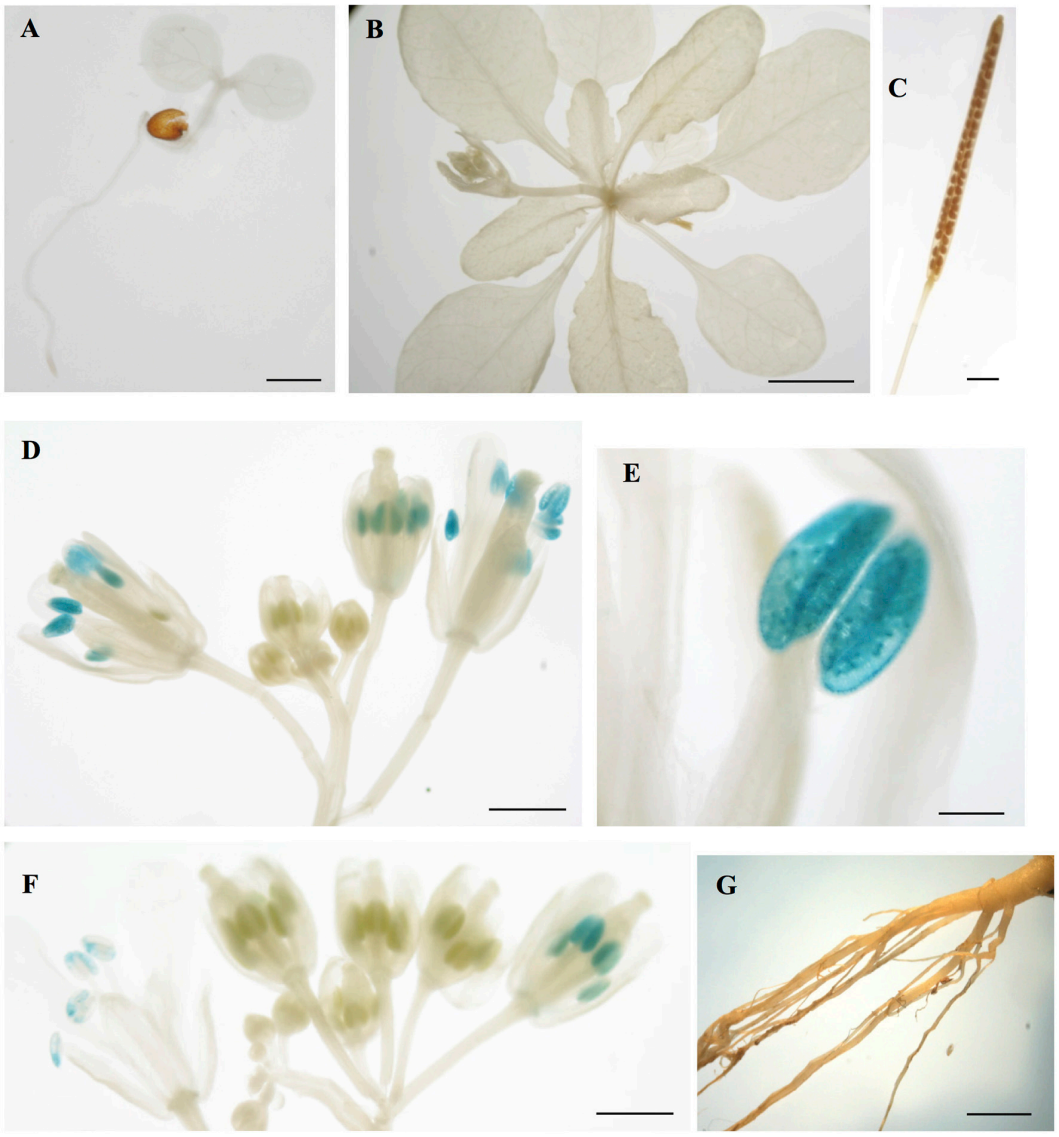
Promoter analysis of *CML15* reveals very specific floral activity. Representative images of histochemical *CML15pro::GUS* reporter activity in *Arabidopsis* tissues, **(A)** 7-day-old seedling, **(B)** 22-day-old rosette, **(C)** mature silique, **(D,F)** stage 6–13 flowers, **(E)** anther at anthesis, and **(G)** 25 day old roots. Images are representative from a minimum of two independent transgenic lines. Bars = 1 mm **(A,C,E,G)**, 0.1 mm **(D)**, 5 mm **(B)**, 2 mm **(F)**.

**Figure 7:**
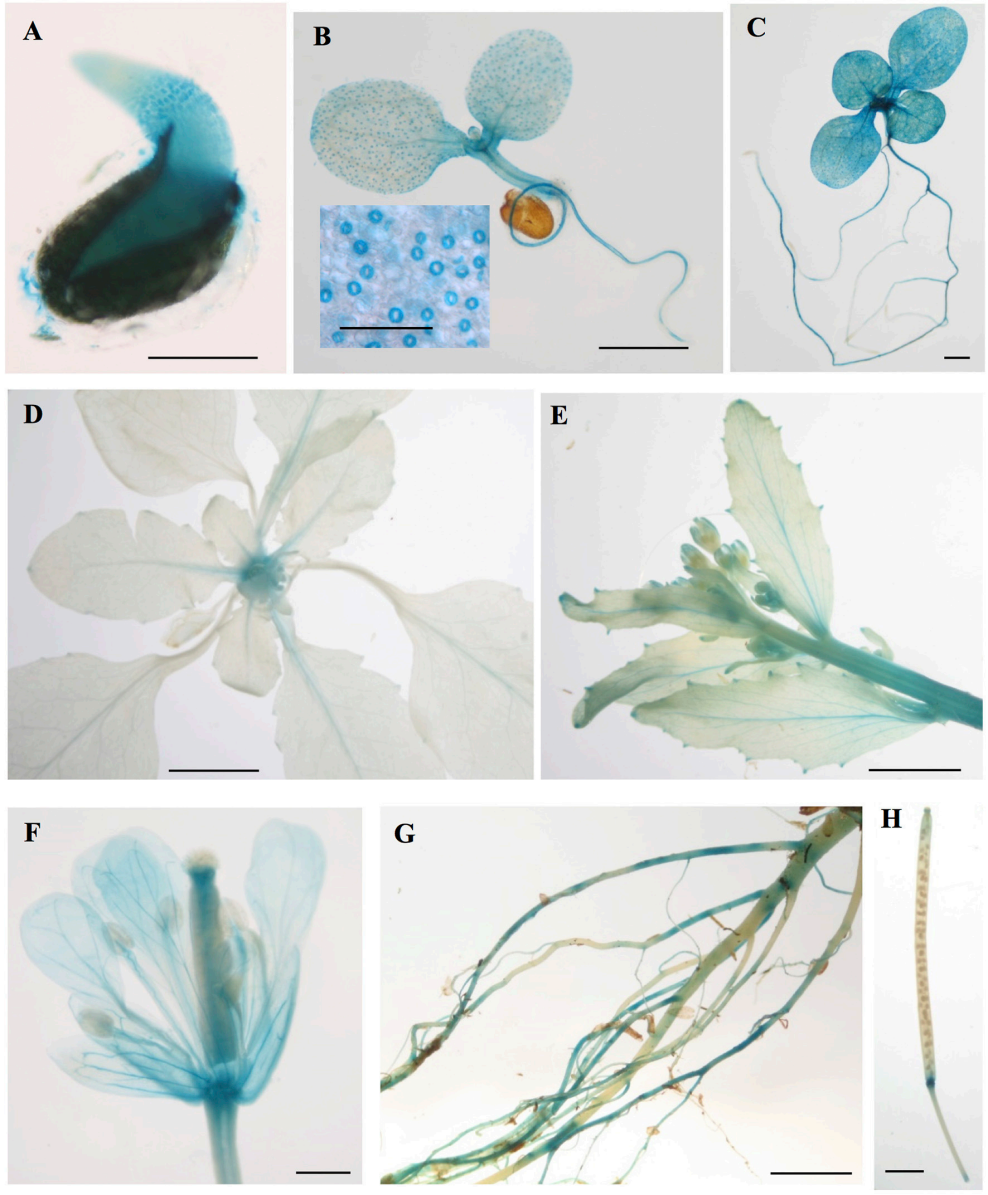
Promoter analysis of *CML16* reveals broad tissue and developmental-stage activity. Representative images of histochemical *CML16pro::GUS* reporter activity in *Arabidopsis* tissues: **(A)** 1-day-old seedling, **(B)** 7-day-old seedling, inset: closeup of guard cells, **(C)** 10-day-old seedling, **(D)** 22-day-old rosette, **(E)** primary inflorescence at 25 days, **(F)** stage ~13 flower, **(G)** mature roots, **(H)** mature silique. Images are representative from a minimum of two independent transgenic lines. *Bars* = 0.2 mm (**A,B** inset), 1 mm **(B,C)**, 5 mm **(D,E)**, 0.5 mm **(F)**, 2 mm **(G,H)**.

**Figure 8:**
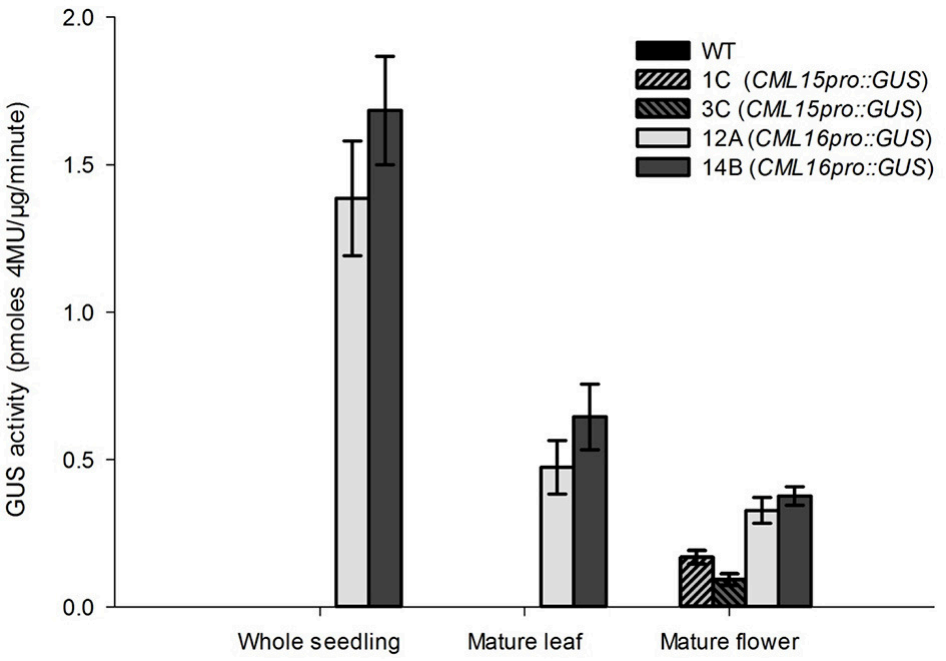
Quantitative fluorometric analysis of *CML15pro::GUS* and *CML16pro::GUS* reporter activity across whole seedling, mature leaf, and mature floral *Arabidopsis* tissues. Specific *CML15pro::GUS* and *CML16pro::GUS* activities were assayed as described in the section Materials and Methods. Specific GUS activity (mean ± SE) is shown for each line. Extracts from WT *Arabidopsis* were assayed as negative controls for background fluorescence and yielded no detectable signal. Annotations 1C, 3C, 12A, 14B, refer to the names of the independent transgenic lines used for analyses.

To gain insight into the tissues and cells types that these CMLs function in, qualitative histochemical *CML-promoter::GUS* reporter analysis was performed to elucidate the spatial patterns of *CML15* and *CML16* promoter activity across a representative set of developmental stages from radical emergence to floral maturation. *CML15pro::GUS* analysis indicates that *CML15* promoter activity is very restricted and was detectable only in mature pollen and anther tissue (Figure 6). In marked contrast, *CML16pro::GUS* analysis revealed strong *CML16* promoter activity across most *Arabidopsis* tissues (Figure 7). *CML16pro::GUS* activity was detected in the root tip of mature embryos, whole 7- and 10-day-old seedlings including guard cells, and the vasculature of adult leaves and inflorescences (Figures 7A–E). In addition, *CML16pro::GUS* activity was observed in vasculature tissue of petals, the stigma, and in the vasculature of filaments in mature (~stage 10–13) floral organs (Figure 7F). Quantitative fluorometric *CML-pro::GUS* analysis using soluble protein extracts from whole seedlings (stage 3), mature leaves (stage 5) and mature floral (stage 7) tissues from *CML15pro::GUS* and *CML16pro::GUS* plants corroborated the qualitative promoter activity data (Figure 8). As demonstrated by the histochemical GUS analysis, *CML15* promoter activity was only found within stage 10–13 floral tissue extract (Figure 8). Conversely, *CML16* promoter activity was detected in all stages assayed for GUS enzyme activity, with the greatest specific activity detected in whole seedling tissue extracts. Taken together, these data suggest a very restricted expression for *CML15* in specific male reproductive tissues compared to broad expression of *CML16* across various tissues and throughout development. Notably, CML16 is largely, if not exclusively, absent from the floral tissue where CML15 was observed suggesting these CMLs have non-overlapping patterns of expression.

## Discussion

Despite representing the largest class of Ca^2+^ sensors in plants, most CMLs remain unstudied. Among the 50 CMLs in *Arabidopsis*, only a few have been examined to date at any biochemical or physiological level. Given the importance of Ca^2+^ signaling in plants, elucidating the properties and roles of plant-specific Ca^2+^ sensors is a necessary step toward a broader understanding of the mechanisms underlying the actions of such a universal second messenger. It has long been hypothesized that the remarkable diversity of Ca^2+^ sensors in plants, and the presence of plant-specific families such as CMLs, reflect the need for plants as sessile organisms to coordinate cellular responses to various stimuli. While such a hypothesis is difficult to test directly, analysis of CMLs provides insight into how they differ from CaM and other Ca^2+^ sensors and therefore contribute to cell signaling in distinct ways.

Our examination of two *Arabidopsis* paralogs, CML15 and CML16, from subgroup 4 indicates that they possess unique biochemical properties in comparison to CaM and other CMLs examined to date. At 74% identity to each other, CML15 and CML16 are closely related and carry the hallmark predicted EF-hands of Ca^2+^ sensors but the variation within these domains (Figure 1), and our biochemical analyses (Figures 2–5), suggest they likely respond to Ca^2+^ signals differently than CaM. Multiple lines of evidence indicate that both CML15 and CML16 possess the general biochemical properties expected of Ca^2+^ sensors. Far-UV CD revealed that both CMLs display notable α-helical character in the absence of Ca^2+^. CML16 shows a modest increase in α-helical character in the presence of Ca^2+^, reminiscent of CaM (Martin and Bayley, 1986), whereas helical content for CML15 undergoes only a slight change upon Ca^2+^ binding. From this data, we cannot conclude that these minor changes in helical content are critical structural responses in these CMLs to Ca^2+^ binding. Importantly, in the case of CaM, the changes in response to Ca^2+^ binding reflect predominantly a reorientation of the helices, as opposed to strictly a change in α-helical content (Finn et al., 1995; Zhang et al., 1995). Likewise, Ca^2+^-CML15 and Ca^2+^-CML16 CD spectra may indicate similar changes in helical orientation to accommodate Ca^2+^-binding and expose regions of hydrophobicity that are likely important for target interaction as is the case for CaM. As downstream targets of CML15 and CML16 have not yet been identified, it is unknown at this point whether these differences in structure contribute to properties such as target specificity. The CD data for CML15, where Ca^2+^ binding appears to have little impact on secondary structure, is reminiscent of CML43 (Dobney et al., 2009) and CML42 (Bender et al., 2014). Interestingly, the conserved CaM, AtCaM1, shows similar behavior when the two N-terminal EF-hands are rendered incapable of binding Ca^2+^ (Astegno et al., 2016). Conversely, the changes in secondary structure of CML16 suggested by CD analysis are similar to previously reported data for CML37 (Scholz et al., 2014), CML39 (Bender et al., 2013) and CML36 (Astegno et al., 2017) where Ca^2+^-binding increases helical content. It is not yet clear how these subtle structural distinctions contribute to the functions of different CMLs but it underscores the biochemical complexity of this large family of Ca^2+^ sensors.

The reversible exposure of hydrophobic regions in response to Ca^2+^ binding is a defining biochemical feature of CaM and is important for target interaction (Ikura and Ames, 2006; Gifford et al., 2007). One of the most striking differences we observed for CML15 and CML16 in comparison to CaM is that both CMLs display substantial hydrophobic exposure even in the absence of Ca^2+^ (Figure 3, Supplementary Figure 1). As a consequence, in the presence of Ca^2+^ and Mg^2+^, CML15 and CML16 display fold-inductions in ANS fluorescence that are markedly lower than that of Ca^2+^-CaM (Supplementary Figure 1). Although both CML15 and CML16 show intrinsic hydrophobicity in the absence of Ca^2+^, importantly, neither undergo a notable increase in hydrophobic exposure in the presence of excess MgCl_2_ alone. This observation is consistent with the hypothesis that these CMLs function as Ca^2+^ sensors in a cellular environment where background Mg^2+^ levels are expected to be much higher than Ca^2+^. It is interesting that while both CML15 and CML16 show greater intrinsic hydrophobicity in the absence of Ca^2+^, the magnitude of change in exposed hydrophobicity upon Ca^2+^ binding is much greater for CaM (Supplementary Figure 1). This suggests that the tertiary structures of the Ca^2+^-bound CMLs have less exposed hydrophobicity relative to CaM. Despite having higher GRAVY scores, the reduced hydrophobic exposure of Ca^2+^-bound forms of CML15 and CML16 compared to CaM might in part be accounted for by their lower relative methionine (Met) content given that Met residues account for 46% of the solvent accessible hydrophobic regions of CaM (O'Neil and DeGrado, 1990; Yuan et al., 1999). Determining the structure of the holo- and apo-forms of these CMLs via x-ray crystallography or NMR is needed to conclusively address these questions. Interestingly, CML15 and CML16 show slightly increased hydrophobic exposure in the presence of EGTA/EDTA (Figure 3). While the mechanism underlying this phenomenon is unclear, it is reminiscent of that observed for the C-domain of rabbit skeletal Ca^2+^ sensor troponin-c which exhibits greater ANS fluorescence in the presence of 0.5 mM EGTA than in 10 mM MgCl_2_ (Grabarek, 2011). An alternatve Ca^2+^-chelator (trisodium citrate) did not evoke this response from CML15 or CML16, suggesting that hydrophobic exposure in the presence of EGTA/EDTA might be an artifact of direct interaction with the CMLs, perhaps mimicking in some respect CML-target interaction, although this remains speculative. Regardless, both CML15 and CML16 show greater intrinsic hydrophobicity than CaM in the absence of Ca^2+^ but retain the ability to respond to Ca^2+^ signals with conformational changes that increase exposed hydrophobic regions, features expected of Ca^2+^ sensors. The levels of exposed hydrophobicity, both in apo- and holo-forms of CMLs vary among family members. For example, CML36 resembles CMl15 and CML16 in that only a modest increase in exposed hydrophobicity is observed upon Ca^2+^ binding (Astegno et al., 2017). In contrast, CML37, CML42, CML43, behave more like CaM, displaying low intrinsic hydrophobic exposure in the apo form and much greater relative increases in response to Ca^2+^ (Dobney et al., 2009; Bender et al., 2014; Scholz et al., 2014). Interestingly, CML14, binds a single Ca^2+^ atom and shows no change in exposed hydrophobicity at all (Vallone et al., 2016). This structural diversity among CMLs is consistent with the hypothesis that they play distinct though possibly overlapping roles, predominantly as Ca^2+^ sensors. The challenge in future work will be to assess the importance of these different structural properties in target binding or other functional contexts.

ITC analysis revealed the Ca^2+^-binding affinities and thermodynamic parameters of CML15 and CML16 and also shed light on the Mg^2+^-binding properties of these CMLs. Importantly, both CMLs are able to bind Ca^2+^ with K_*d*_ values in the high nanomolar to low μM range in a mM Mg^2+^ background (Table 1), consistent with what would be considered physiologically relevant Ca^2+^ levels (Rizzuto and Pozzan, 2006; Dodd et al., 2010; Steinhorst and Kudla, 2014), thereby supporting their predicted roles as Ca^2+^ sensors. Various environmental stimuli induce cytosolic Ca^2+^ fluxes in plant cells that reach into the μM range and microdomains near Ca^2+^ channels are expected to spike at levels in the tens of μM (Rizzuto and Pozzan, 2006; Steinhorst and Kudla, 2014). Having a repertoire of Ca^2+^ sensors tuned to a range of Ca^2+^ signals is likely a key aspect of successful information processing in plants. As might be expected for a large family of Ca^2+^ sensors, the Ca^2+^ affinities among CMLs tested to date vary but generally fall within what would be considered a physiological range (nM–μM). The Ca^2+^ dissociation values for CML15 and CML16 are comparable to those observed for CML42 (Dobney et al., 2009), CML43 (Bender et al., 2014), CML14 (Vallone et al., 2016), and CML36 (Astegno et al., 2017). The presence of both high- and low-affinity Ca^2+^ sites in CMLs appears to be an emerging pattern (Bender et al., 2014; Astegno et al., 2017) but how these features relate to *in vivo* function remains unclear. A recent study on a tobacco CML, rgsCaM, revealed a low affinity for Ca^2+^ *in vitro*, calling into question whether rgsCaM could function as a Ca^2+^ sensor *in vivo* (Makiyama et al., 2016). These authors speculate that target binding might enhance the affinity of rgsCaM for Ca^2+^ as has been observed for CaM (Gifford et al., 2007).

It is noteworthy that while sequence analysis predicts four EF hands for both CMLs, ITC indicates two and three Ca^2+^ binding events for CML15 and CML16, respectively. This is reminiscent of the differences between predicted and observed binding sites noted for other CMLs (Dobney et al., 2009; Bender et al., 2014; Vallone et al., 2016). However, we cannot exclude the possibility that, despite extensive dialysis against EGTA, some Ca^2+^ remained bound to these CMLs throughout the purification process and led to underestimations in binding site numbers. Nevertheless, as discussed above, the variations in the EF-hand loops and nearby regions of these CMLs indicates they likely possess Ca^2+^ binding properties that differ from those of CaM. Synthetic CaM (SynCaM), a hybrid of mammalian and plant CaM, exhibits two exothermic (enthalpically-driven) binding events followed by two endothermic (entropically-driven) Ca^2+^-binding events (Gilli et al., 1998). Interestingly, ITC data demonstrates that for CML15, the Ca^2+^-binding event that occurs first is primarily entropically driven (site 1), and the second Ca^2+^-binding event is enthalpically driven (Table 2). In the absence of a strong entropic contribution to the first binding event we would expect the interaction with the more exothermic enthalpy to occur first. However, the entropic contribution to site-1 Ca^2+^ binding is sufficiently strong in the presence of Mg^2+^ to generate a more negative free energy change than for the second binding event, thereby driving site-1 Ca^2+^-binding to occur first. Interestingly, the titration of CML15 with Mg^2+^ generates an endothermic Mg^2+^-binding event, indicating this event is entropically driven. This phenomenon is unusual, as electrostatic attraction (favorable enthalpy) typically drives ion binding (Linse et al., 1991; Gifford et al., 2013). As a result, positive entropy is responsible for driving Mg^2+^ binding to CML15. Interestingly, the N-terminal region of the Ca^2+^-binding domain of NADPH oxidase 5 (NCaBD of NOX5) generated a similar ITC isotherm to that of CML15 titrated with Ca^2+^ (Linse and Chazin, 1995; Wei et al., 2012). ITC analysis of EF-hand I and EF-hand II of NCaBD suggests that EF-hand II has the higher affinity for Ca^2+^, triggering conformational changes that promote the binding of Ca^2+^ to EF-hand I. Like CaM, these Ca^2+^-binding sites in NCaBD display positive cooperativity (Wei et al., 2012). As proteins with considerable structural similarity to CaM, it is reasonable to speculate that Ca^2+^ binding to CML15 and CML16 occurs with positive cooperativity, but a more detailed structural analysis is needed to empirically assess this possibility.

**Table 2:** Thermodynamic parameters and dissociation values^a^^,^^b^ for Mg^2+^-binding to CML15 and CML16 in a Ca^2+^-free background.

	**K*_*d*_* (μM)**	**ΔH (kJ mol^−1^)**	**ΔS (J K^−1^ mol^−1^)**
CML15	6.95 ± 0.35	7.52 ± 0.43	135.35
CML16	0.03 ± 0.01	−8.43 ± 2.34	53.89

a *Mean ± SE*.

b *Stochiometries cannot be accurately determined from monophasic isotherms and values represent macroscopic K_d_*.

In the case of CML16, one of the Ca^2+^-binding sites was only detectable in the absence of Mg^2+^. Collectively, the ITC data for CML16 suggests that Mg^2+^ binding might entirely preclude Ca^2+^ binding to one of the EF-hands. Without additional structural information, it is unclear how Mg^2+^ would affect Ca^2+^ binding to CML16. *In vivo*, [Mg^2+^]_cyt_ is likely about 3 orders of magnitude greater than [Ca^2+^]_cyt_ which is estimated to be in the 100–200 nM range (Waters, 2011), making possible competition or allosteric effects of Mg^2+^ potentially more relevant. It should also be noted that there may be multiple weak Mg^2+^ binding sites on these CMLs that led to our underestimating Mg^2+^ K_*d*_ values using a single-site binding model. Weaker Ca^2+^ affinities in the presence of Mg^2+^ are common for EF-hand containing proteins based on suggestions that Mg^2+^ might be a direct competitor for Ca^2+^ binding to EF-hands (Ohki et al., 1997; Malmendal et al., 1999; Clapham, 2007; Gifford et al., 2007). The isotherms for the Ca^2+^ titration of CML16 are remarkably similar to those observed for GmCaM4, a soybean CML with 77% identity to mammalian CaM (Gifford et al., 2013). In the case of GmCaM4, Mg^2+^ stabilizes the closed conformation and acts as a competitive antagonist, decreasing the affinity for Ca^2+^ by ~6.3-fold. We speculate that Mg^2+^ fulfills a similar role for CML16 resulting in a lag in saturation for the titration of CML16 with Ca^2+^, and a decrease in the affinity of CML16 for Ca^2+^. Interestingly, Mg^2+^ binding to the N-terminal domain of GmCaM4 has been suggested to increase the affinity of the EF-hands for Ca^2+^ by folding the domains, and thus paying the energetic costs of restructuring the EF-hands (Gifford et al., 2013). This means that Mg^2+^ serves as both a competitive antagonist and an allosteric activator for GmCaM4. As mentioned, quantitatively similar changes in α-helical character occur at the level of secondary structure for CML16 in the presence of Ca^2+^ and in the presence of Mg^2+^. CD data shows that CML16 exhibits comparable increases in α-helical character in the presence of Ca^2+^ or Mg^2+^. Taken together, this data indicates that Mg^2+^ may play the role of an allosteric activator for CML16 but, as noted above, Mg^2+^ does not induce conformational changes that lead to hydrophobic exposure for either CML15 or CML16 (Figure 3, Supplementary Figure 2) and thus would not be expected to substitute for Ca^2+^ in target interaction.

Aside from EF-loop I, which is the most conserved among the predicted EF hands in comparison to CaM (Figure 1), it is difficult to speculate which residue changes in the CMLs are responsible for the differences in metal-binding properties relative to CaM. Moreover, it remains possible that regions outside of the EF hands themselves contribute to structural differences that impact cation binding. Regardless, the picture that emerges is that although both CML15 and CML16 are biochemically distinct from CaM, these CMLs possess high-affinity Ca^2+^ binding sites and respond to Ca^2+^ binding, but not Mg^2+^ binding, with substantial conformational changes that expose hydrophobic regions. The latter property is likely critical in considering these CMLs as *bona fide* Ca^2+^ sensors whose postulated roles are to regulate downstream targets. How the biophysical differences from CaM contribute to CML physiological function remains an open question for further study. In future, identification of targets for these CMLs will be important to assess how CML-target interaction is affected by cation binding and *vice versa*. Moreover, caution is always merited in trying to extrapolate the *in vitro* biochemical properties of Ca^2+^ sensors to their behavior under cellular conditions.

The very different patterns of *CML15* and *CML16* promoter activity we observed suggest that spatial expression of these genes likely overlap little, if at all, in terms of their tissue distribution. The broad pattern of *CML16pro::GUS* activity across a range of tissues and developmental stages is in marked contrast to that of *CML15pro::GUS* which was observed exclusively in floral tissue, specifically mature anthers and developing pollen grains. While *CML16pro*::*GUS* activity was also detected in floral tissue, it was excluded from the anthers where *CML15pro*::*GUS* activity was quite strong. These data suggest that *CML15* and *CML16* likely play distinct roles in floral reproductive structures. The expression of *CML15* in pollen is consistent with the fact that Ca^2+^ signaling is very important in pollen germination and tube growth and a number of Ca^2+^ sensors have been implicated in these processes (Steinhorst and Kudla, 2013). The restriction of *CML15* promoter activity to anther and pollen tissue is reminiscent of *CML39* (Vanderbeld and Snedden, 2007). However, *CML39* expression is strongly inducible across a range of tissues by abiotic stress and the hormone methyl jasmonate (Vanderbeld and Snedden, 2007). In contrast, we did not observe any induction of *CML15* promoter activity when plants were exposed to different abiotic stress conditions (data not shown) suggesting the role of CML15 is primarily associated with male floral organ development.

It is interesting to note that *CML16* promoter activity was strong in guard cells in seedlings but not mature plants, suggesting a role for CML16 in early guard cell development. This pattern is similar to that reported previously for CML38 (Vanderbeld and Snedden, 2007). As the gatekeepers of CO_2_ entry and water vapor exit, guard cells play key roles in both water relations and carbon fixation by adjusting their turgidity and thereby controlling stomatal pore aperture. Ca^2+^ is well-established as a key second messenger in guard cell signaling and the strong activity of *CML16pro::GUS* in these cells suggests that CML16 may be serving as a Ca^2+^ sensor in these cells during stomatal pore opening and/or closing, both of which involve Ca^2+^ signals (Murata et al., 2015; Agurla and Raghavendra, 2016). *CML16pro::GUS* activity was also strong in vascular tissues at all growth stages examined, indicating that *CML16* may participate in vascular tissue development or function.

To date, information on *CML* expression patterns comes predominantly from global transcriptomic analyses although there have been a number of promoter-reporter studies. For Arabidopsis, transcriptome database mining predicted that *CaMs* are broadly expressed across tissues and developmental stages whereas *CMLs* cluster into five main expression groups that range from highly restrictive to widespread like the *CaMs* (McCormack et al., 2005). Likewise, transcriptome analysis for soybean *CMLs* indicates similar expression-based clustering with many displaying broad tissue distribution and at least 7 *CMLs* being floral specific (Zeng et al., 2017). Promoter-reporter analysis has corroborated or expanded upon these data for a few Arabidopsis *CMLs*. For example, *CML42* shows broad expression across a wide range of tissues and developmental stages whereas its close paralog, *CML43*, is restricted to root tips under normal growth conditions (Dobney et al., 2009; Bender et al., 2014). This contrast in expression patterns between paralogs has been observed for other CMLs. *CML39* is expressed predominantly in male floral tissue whereas *CML38* shows much broader tissue expression (Vanderbeld and Snedden, 2007). These examples are thus reminiscent of the patterns observed in the present study for *CML15* and *CML16*. While our GUS reporter data agree well with the expression patterns for *CML15* and *CML16* as suggested by public transcriptome databases (e.g., bar.utoronto.ca (Toufighi et al., 2005), several caveats should be kept in mind when interpreting *pro::GUS* expression data. Although both *CML15* and *CML16* are intronless genes, we cannot exclude the possibility that some regulatory elements may be absent from the genomic regions we used for analysis of *CML* promoter activity. In addition, GUS enzyme activity is not a direct indicator of transcription or translation rates or protein stability. Nevertheless, our GUS reporter analysis has provided new insight into *CML15* and *CML16* tissue- and cell-specific promoter activity and emphasized the sharp differences in spatial expression for these two genes. Although reverse genetic approaches, such as gene-knockout analysis, often provide insight into gene/protein function, we did not observe any phenotypic differences using T-DNA insertional knockout lines for CML15, CML16, or with CML15/16 double-knockout lines (data not shown). Functional redundancy among the large CML family is the most likely explanation for the absence of detectable phenotypes in these mutants. However, it remains likely that under some specific environmental condition the loss of these CMLs would manifest itself in a fitness cost or aberrant phenotype; we simply don't yet know what such conditions would be. Generation of higher order mutants of multiple CML knockouts in future studies may help unravel the role of CML15 and CML16 in plant development.

It is necessary to also point out that the subcellular localizations of CML15 and CML16 have not been empirically demonstrated. However, it seems highly likely that both of these proteins are cytosolic given that (i) neither possesses an N- or C-terminal extension relative to CaM that might function as a localization sorting sequence, and (ii) prediction algorithms such as found at Suba4 (suba.live, Hooper et al., 2017) and Aramemnon (aramemnon.uni-koeln.de/multi-prediction, Schwacke et al., 2003) present consensus predictions for cytosolic localization with high probability.

The breadth of biochemical properties, tissue distributions, and target interactions among Ca^2+^ sensors, including CMLs, almost certainly reflects both the importance and complexity of Ca^2+^ signaling in plants. Going forward, future analysis aimed at identifying downstream targets of CML15 and CML16 is needed in order to delineate the Ca^2+^ signaling pathways and cellular events that these proteins participate in. Target identification will also allow for a comparison with CaM of the structural mechanisms through which these Ca^2+^ sensors interact with their effectors. It is important to continue investigations into the properties and functions of CMLs as a unique and highly diverse family of Ca^2+^ sensors as it brings us closer to a more complete picture of Ca^2+^ signaling in plants and of information processing in general.

## Author contributions

WS: designed the experiments and supervised the study; AO, AD, MU, and KM performed the experiments with assistance from WS. All authors analyzed, interpreted, and discussed the results and contributed to writing of the manuscript.

### Conflict of interest statement

The authors declare that the research was conducted in the absence of any commercial or financial relationships that could be construed as a potential conflict of interest.

## Supplementary material

Supplementary Figure 1 Ca^2+^- and Mg^2+^-induced changes in exposed hydrophobicity of an evolutionarily-conserved recombinant CaM (CaM81) as demonstrated by ANS fluorescence. Fluorometric scans (430–600 nm) were recorded following the addition of 15 μM CaM to 250 μM ANS where all conditions used ANS buffer (10 mM Tris-Cl, pH 7.5, 100 mM KCl and 1 mM DTT). The *y*-axis depicts fluorescence in international arbitrary units (IAU). Fluorescence values were analyzed relative to a protein-free ANS control sample that was subtracted from the data as background. Each data set is representative of five experimental replicates.

Supplementary Figure 2 Hydrophobic (phenyl-sepharose) column chromatography of recombinant CML15 **(A)** and CML16 **(B)** as visualized by Coomassie-stained SDS-PAGE. Chromatography was performed in the presence of either 1 mM CaCl_2_, 1 mM CaCl_2_ and 1 mM MgCl_2_, 1 mM MgCl_2_, or 1 mM EGTA, as indicated. All column-binding events were followed with a subsequent wash step using 10 bed-volumes of 0.2 M KCl (“W1-W10”), and a wash step using 10 bed-volumes with 0.4 M KCl (“W11-W20”). “UB” denotes the starting protein sample that was loaded onto the column, “FT” denotes the unbound protein flow-through, and “E” denotes eluted fractions.

Supplementary Figure 3 Homology models of CML15 and CML16 based on a Ca^2+^-CaM crystal structure. Models were generated using Phyre2.0 software and PyMol molecular graphics system as described in the section Materials and Methods. The structure of *Paramecium tetraurelia* CaM (UNIPROT identifier P07463) was selected by Phyre2.0 as the CaM with the greatest sequence identity in the structural database for comparison to CML15 (UNIPROT identifier Q9FZ75) and CML16 (UNIPROT identifier Q9LI84). Hydrophobic residues are shaded gray, polar but uncharged residues are pale cyan, charged residues are blue, prolines are magenta, cysteines are green, and Ca^2+^ atoms are presented as green spheres in the CaM model. The grand average of hydropathy (GRAVY) score was determined as described in the section Materials and Methods and is presented below each model.

Supplementary Table 1 Oligonucleotide primers used for PCR.
