# Supplementary figures and images for: Arabidopsis Calmodulin-Like Proteins, CML15 and CML16 Possess Biochemical Properties Distinct from Calmodulin and Show Non-overlapping Tissue Expression Patterns
Source: Front Plant Sci. 2017 Dec 22;8:2175. doi: 10.3389/fpls.2017.02175 (PMC5743801; doi:10.3389/fpls.2017.02175)

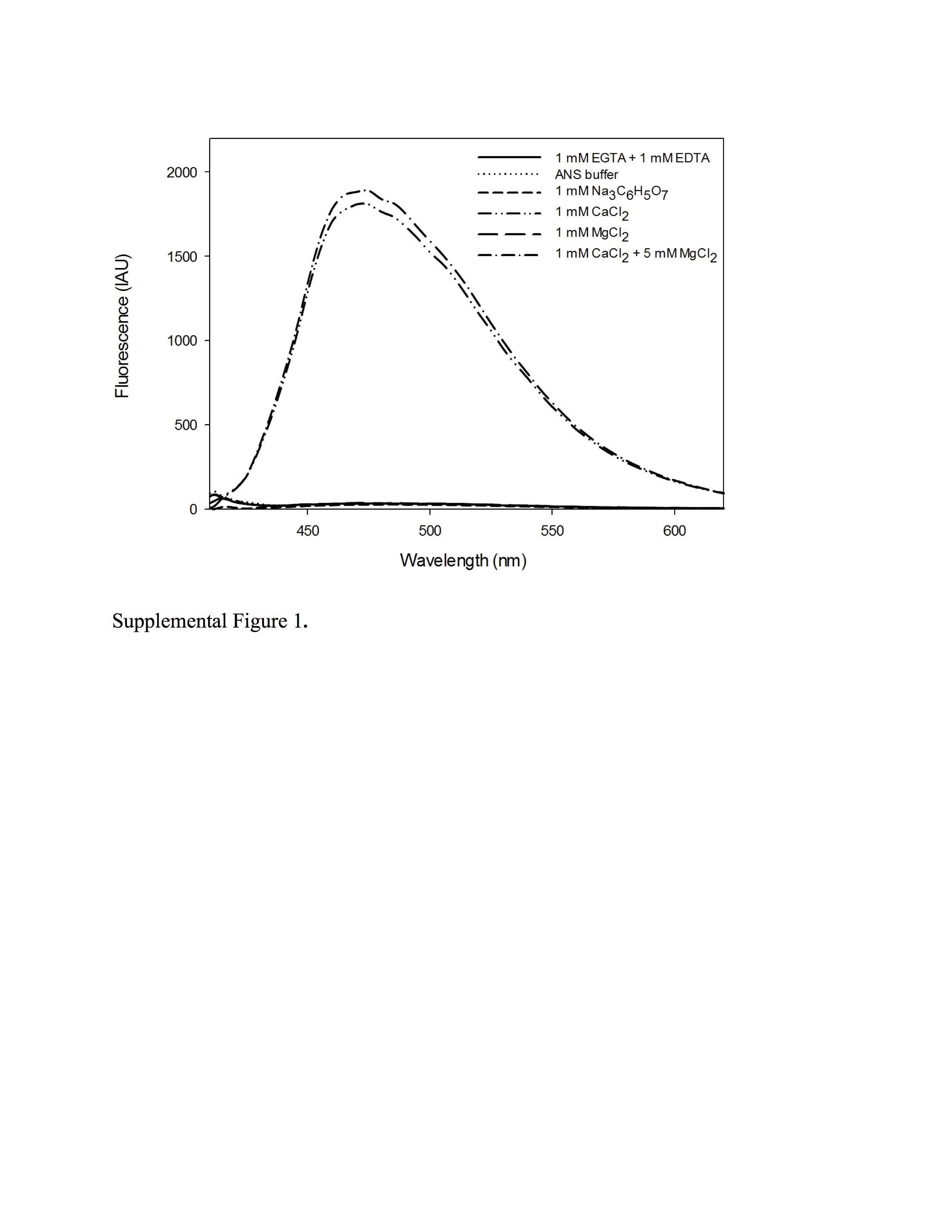

Supplement: Supplementary Figure 1 — Ca2+- and Mg2+-induced changes in exposed hydrophobicity of an evolutionarily-conserved recombinant CaM (CaM81) as demonstrated by ANS fluorescence. Fluorometric scans (430–600 nm) were recorded following the addition of 15 μM CaM to 250 μM ANS where all conditions used ANS buffer (10 mM Tris-Cl, pH 7.5, 100 mM KCl and 1 mM DTT). The y-axis depicts fluorescence in international arbitrary units (IAU). Fluorescence values were analyzed relative to a protein-free ANS control sample that was subtracted from the data as background. Each data set is representative of five experimental replicates. [file Image1.JPEG]

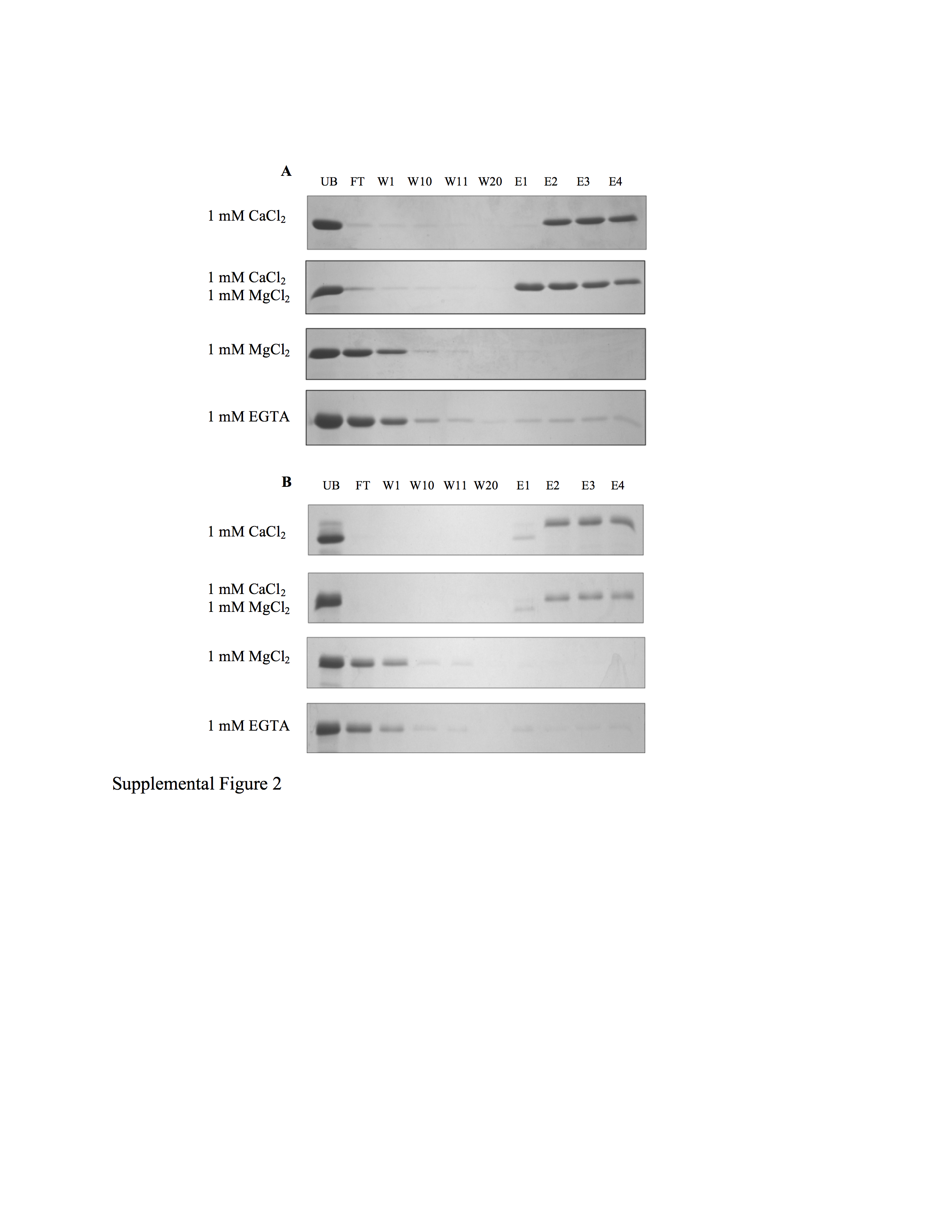

Supplement: Supplementary Figure 2 — Hydrophobic (phenyl-sepharose) column chromatography of recombinant CML15 (A) and CML16 (B) as visualized by Coomassie-stained SDS-PAGE. Chromatography was performed in the presence of either 1 mM CaCl2, 1 mM CaCl2 and 1 mM MgCl2, 1 mM MgCl2, or 1 mM EGTA, as indicated. All column-binding events were followed with a subsequent wash step using 10 bed-volumes of 0.2 M KCl (“W1-W10”), and a wash step using 10 bed-volumes with 0.4 M KCl (“W11-W20”). “UB” denotes the starting protein sample that was loaded onto the column, “FT” denotes the unbound protein flow-through, and “E” denotes eluted fractions. [file Image2.JPEG]
