# Supplementary material for: Arabidopsis Calmodulin-Like Proteins, CML15 and CML16 Possess Biochemical Properties Distinct from Calmodulin and Show Non-overlapping Tissue Expression Patterns
Source: Front Plant Sci. 2017 Dec 22;8:2175. doi: 10.3389/fpls.2017.02175 (PMC5743801; doi:10.3389/fpls.2017.02175)
Supplement: Supplementary Table 1 — Oligonucleotide primers used for PCR. [file Table1.docx]

Supplementary Table 1. Oligonucleotide primers used for PCR

| Primer Name | Primer Sequence (5’-3’) |
| --- | --- |
| CML15PRO-F | AAGCTTAACAACTCAATTTCAGTTTCTTG |
| CML15PRO-R | GGATCCTTTAGCAACACAAATAAAAATCG |
| CML16PRO-F | AAGCTTATTAATGACATAAAAGTTTCCAC |
| CML16PRO-R | GGATCCCTTAAACTCGAAGAAACC |
|  |  |
| CML15-F | CATATGGAGGATCAGATAAGACAACTT |
| CML15-R | GCATAAGAAATGATCACGAATTAAT |
| CML16-F | CATATGGCGTCAACAAAACCAACCG |
| CML16-R | GATCAAGAAGCGGTTAATCCAAG |
